# Exploring the barriers to cervical screening and perspectives on new self-sampling methods amongst under-served groups

**DOI:** 10.1186/s12913-024-12098-2

**Published:** 2025-01-15

**Authors:** Stephanie Gillibrand, Helen Gibson, Kelly Howells, Sean Urwin, Jennifer C. Davies, Emma J. Crosbie, Caroline Sanders

**Affiliations:** 1 Centre for Primary Care & Health Services Research, School of Health Sciences, Faculty of Biology, Medicine & Health, The University of Manchester, Greater Manchester, England UK; 2 NIHR Greater Manchester Patient Safety Research Collaboration, Centre for Primary Care & Health Services Research, School of Health Sciences, Faculty of Biology, Medicine & Health, The University of Manchester, Greater Manchester, England UK; 3 Health Organisation, Policy and Economics, Centre for Primary Care & Health Services Research, School of Health Sciences, Faculty of Biology, Medicine & Health, The University of Manchester, Greater Manchester, England UK; 4 Gynaecological Oncology Research Group, Division of Cancer Sciences, Faculty of Biology, Medicine & Health, The University of Manchester, Greater Manchester, UK; 5 Department of Obstetrics and Gynaecology, St Mary’s Hospital’, Manchester University NHS Foundation Trust, Manchester Academic Health Science Centre, Manchester, UK; 6 Gynaecological Oncology, Gynaecological Oncology Research Group, Division of Cancer Sciences, Faculty of Biology, Medicine a& Health, The University of Manchester, Greater Manchester, UK; 7 NIHR Greater Manchester Patient Safety Research Collaboration, Centre for Primary Care & Health Services Research, School of Health Sciences, Faculty of Biology, Medicine & Health, The University of Manchester, Greater Manchester, England UK; 8 NIHR Applied Research Collaboration for Greater Manchester (ARC-GM), Greater Manchester, UK

**Keywords:** Cervical screening, Self-sampling, Under-served groups, Intersectionality

## Abstract

**Background:**

Cervical screening rates have fallen in recent years in the UK, representing a health inequity for some under-served groups. Self-sampling alternatives to cervical screening may be useful where certain barriers prohibit access to routine cervical screening. However, there is limited evidence on whether self-sampling methods address known barriers to cervical screening and subsequently increase uptake amongst under-screened groups. Addressing this research gap, the study aims to understand experiences during and barriers to attending cervical screening for under-screened groups and; explore the views of individuals eligible for screening towards self-sampling (vaginal swabbing and urine sampling) as alternative screening methods and how this may address existing barriers to screening.

**Methods:**

We draw on three integrated theoretical frameworks (access to primary care services, intersectional and feminist perspectives) to examine participants’ barriers to screening and views toward self-sampling methods. We undertook primary qualitative data collection (interviews and focus groups) with 46 participants, facilitated by collaborations with the VCSE sector which successfully enhanced reach to under-served communities.

**Results:**

Known barriers to cervical screening persist for under-screened participant groups, but we also find numerous examples of good practice where some participants’ needs were met throughout the screening process. Both positive and negative experiences tend to centre around experiences with healthcare professionals, with negative experiences also centring around the use of the speculum. Self-sampling methods (vaginal swab and urine collection) were positively received by participants, and may address some existing barriers through the proponents of enhanced choice – between method and location (which also dovetailed with convenience) leading to greater empowerment. The removal of the speculum and lack of invasive examination by a healthcare professional was also positively received.

**Conclusions:**

Whilst barriers to cervical screening remain for under-served groups, examples of good practice are prevalent. Such examples should be implemented more widely to ensure consistency in patient experience and to ensure needs are better met for under-served groups. The introduction of self-sampling alongside traditional methods may reduce barriers to screening, and may boost screening rates for under-screened groups but only if they are implemented with appropriate information and sufficient communication. Failure to implement self-sampling without these considerations may threaten to undermine the identified and important benefits of self-sampling methods.

**Supplementary Information:**

The online version contains supplementary material available at 10.1186/s12913-024-12098-2.

## Background

Cervical screening is a key part of preventative primary care, playing a crucial role in protecting against cervical cancer, which in recent years, accounts for 2% of new cancer cases in females in the UK [1]. Cervical screening (testing of HPV) is available to women and people with a cervix aged 25 to 64 in England. The first invitation is sent (usually via mail) to eligible people at the age of 24.5 years. People aged 25 to 49 receive invitations every 3 years (unless abnormal cells are found after a positive HPV test, in which case they are invited annually). People aged 50 to 64 receive invitations every 5 years [2].

Although incidence of cervical cancer in the UK has reduced by 25% since the 1990s [1], more recently cervical screening coverage has fallen, especially since the onset of the COVID-19 pandemic [3, 4] [5, 6]. For the year 2023–24, 69% of eligible people were screened in England [7]. In particular, certain under-served groups are less likely to attend screening than others. In the UK, under-screened groups include ethnic minority communities, older and younger groups [8], [9], [10] [11], people who live in socially deprived areas [12], those with lower education levels [13], and people with intellectual disabilities [14] [15]. Research has also found that women who have experienced sexual violence are less likely to attend regular screening due in part to the invasive procedure associated with the test [16], and this is also true for women who have experienced homelessness and sexual violence [17].

GP practices in England receive payments as part of the Quality and Outcome Framework (QOF) related to the proportion of their relevant patients who have been screened. More recently there are renewed public health awareness campaigns about screening for cervical cancer [18], alongside the pledged commitment from the NHS to eliminate cervical cancer in the UK by 2040 [19].

Barriers to cervical screening are well documented, with a number of factors contributing to individuals’ decisions to decline attendance. These include, a lack of knowledge and awareness [8, 20–22], embarrassment, and the fear and possible discomfort and/or pain associated with the speculum examination [8, 9] as well as wider structural, practical and logistical barriers including childcare responsibilities and inflexible working arrangements [20, 23–25]. These barriers to cervical screening mirror other known barriers that patients face in accessing primary care services, including availability, timeliness and convenience of primary care services, communication and other inter-personal factors such as continuity of care, and provider and patient attitudes [26–28]. However, within the broader academic literature, the barriers to cervical screening for under-screened groups in England have not been explored through the lens of wider frameworks on access to healthcare services, in particular those relevant to primary care (e.g. candidacy [29], equity of access [30]).

Self-sampling alternatives to cervical screening may help overcome certain barriers that prohibit access to routine cervical screening. Self-samples have similar sensitivity for cervical pre-cancer detection as clinician-obtained cervical samples [31–33] and would achieve substantial savings for the NHS if rolled out in the UK [34]. However, there is limited evidence as to whether self-sampling methods address known barriers to accessing screening and increase uptake amongst under-screened groups (as described above). In the paper, we address this research gap by examining the barriers to screening for under-screened groups in Greater Manchester and explore views about self-sampling (urine sampling^1^ and self-swabbing) within broader frameworks of access to primary care services, intersectional and feminist perspectives. We conclude that there are inconsistencies across participants’ experiences of accessing screening and enduring barriers to screening may have significant implications for under-screened groups, along pre-existing lines of inequalities. New self-sampling methods may address some of these barriers due to offering choice and a sense of greater empowerment. We therefore theorise that self-sampling may boost screening rates for under-screened groups by virtue of enhancing choice between screening methods, which aligns to their identified needs.

## Situating cervical screening within feminist theory

Scholars have previously situated cervical screening programmes in critical feminist perspectives, examining the discursive underpinnings of the policy [35] [36], and the processes that form part of the programme, including invitation based setup and a focus on levels of attenders and non-attenders [37]. It has been claimed that such programmes exert control over women’s autonomy amounting to ‘surveillance’ over their bodies [38] [35] and in this way has become a normalised obligation for women [39] [35].

Bush discusses feminist concepts of resistance towards cervical screening discourses. The impetus for control over the screening process is central here, identifying a lack of choice in being screened, concluding “campaigners for women’s health must debate how a women can make an informed choice about having a smear test…women shouldn’t be having smear tests because they see it as being part of being a woman, but because they have decided – with knowledge of both the advantages and disadvantages of the cervical screening programme – that it’s something that they personally want to do” [35] p442). Such perspectives echo broader feminist literature on women’s sexual and reproductive health, most notably the *Our Bodies Ourselves* campaign [40]: a grassroots effort to centre women and their experiences in public health discourse. Other literature related to female reproductive healthcare has identified that stigmatisation and normatively situated discourses characterise women’s interaction with healthcare and healthcare professionals (HCPs), rooted in gendered asymmetrical power relations [41] [42, 43]. Overall, this literature illustrates the need to centre feminist perspectives in the discussion of women’s reproductive health, policy and practice. We draw on these feminist perspectives to situate our findings within key features of cervical screening: choice, control, knowledge and power.

## Access to cervical screening as ‘human fit’

We draw on theories of access to contextualise our findings exploring the barriers to screening and responses to new screening methods. Voorhees’ et al. [44] framework of ‘access as human fit’ builds on Levesque’s et al. [45] conceptualisation of the five dimensions of access: approachability; acceptability; availability and accommodation; affordability; and appropriateness. In Levesque’s et al. framework, access is defined as “the opportunity to identify healthcare needs, to seek healthcare services, to reach, to obtain or use health care services and to actually have the need for services fulfilled” [45]p9). Voorhees’ adaptation of this framework sees that access to healthcare may be viewed through a lens which centres a multitude of factors from both the perspectives of patients and service users, the healthcare system, and broader contextual factors [44]. This model as ‘access as human fit’ identifies the central features of access as the negotiation of the capabilities and capacity of the healthcare workforce, patient need and human interaction factors within these domains. We draw on this theory of access model as we consider it especially relevant to cervical screening in emphasising subjective relational factors which may influence access. Such factors are apparent at the organisation and institutional level, and between patient and providers, where the capacity and capabilities of the workforce is as important as patient ability to navigate such structures. In addition, the wider context of the healthcare service is especially relevant, such as the post-pandemic context of primary care services and the impact on cervical screening rates [3, 5]. The themes identified in this paper more specifically illustrate how human factors are associated with access. This includes the experiences of patients, interpersonal communication and attitudes (as human interaction between patients and workforce), and patient/population empowerment as contributory domains to an overall model of access as contingent on the fit to human needs and abilities [44].

This study aims to understand experiences during and barriers to attending cervical screening for under-screened groups and; explore the views of individuals eligible for screening towards self-sampling as alternative screening methods and how this may address existing barriers to screening. We draw feminist approaches together with the theory of ‘access as human fit’ to allow feminist principles around the normatively situated navigation of patient/provider interaction and interpersonal power relations in the context of access to cervical screening to be foregrounded. Moreover, normatively situated discourses around obligation are situated alongside the proponents of the theory of access as human fit, namely that this negotiation is dependent upon the organisational and institutional capacity to accommodate (and have the willingness) *as well as* patient autonomy to subvert power-based discourses in the organisation, implementation and practice of screening. Whilst recognising feminist research as an influential background in addition to the theory of ‘access as human fit’, we overlay these frameworks with an intersectional lens. Intersectionality describes the ways in which prevailing power structures converge to maintain forms of oppression and marginalisation, whereby individuals experience intersecting forms of oppression on the basis of these hierarchical social structures [46]. An intersectionality approach allows consideration of the barriers in accessing healthcare, along the lines of these existing forms of marginalisation [47]. We demonstrate how multiple intersecting identities and systems of privilege and oppression interact and can be situated within access as human fit. This permits the complexities of interacting forms of marginalisation to illuminate the multivariable barriers to screening, and by doing so enables consideration of how new screening methods may address such multivariable factors, from both supply and demand. We therefore overlay an intersectionality approach on assessing the barriers to cervical screening and the emergence of new self-sampling methods within the context of access as human fit.

## Methods

### Design

The study utilised a participatory approach, working in partnership with community stakeholders [48]. A project advisory group was set up early in the project’s inception, made up of members of diverse local communities, and members of the wider project team who represent voluntary and community organisations. Interviews and focus groups were used to examine the barriers to screening and views towards self-sampling methods amongst under-screened groups.

### Participants and recruitment

A purposive sampling strategy was used to engage people from under-screened groups, which we define as those identified in the literature as less likely to take up screening. We aimed to recruit 35–45 patient participants. We chose a broad target sample to allow for a wide breadth of diversity, and representation and intersecting identities amongst under-screened groups. Patient participants included younger individuals (< 30); individuals with lived experience of homelessness; those who undertake or have previously undertaken sex work; those with pre-existing mental health or physical health conditions; neurodiverse people, asylum seekers, refugees or those with an insecure migration status. We also sought to engage individuals who have lower rates of re-engagement with screening (i.e. failure to attend follow-up screening). Overall, 46% (*n* = 21) of the sample had attended cervical screening and/or could be considered ‘regular attenders’; just under one third (*n* = 15) had never attended, had missed at least one, and/or were not planning to attend again; and 9 participants did not discuss their screening status.^2^ Screening status was identified from contextual descriptions in the interview and focus group data. In total 46 individuals from under screened groups participated.

The study achieved a diverse sample of participants, and diversity was monitored via participant completion of a demographic information sheet. Key characteristics of the sample are provided in Table 1 below.

**Table 1:** Sample characteristics of patient participants^a^

	**TOTAL (n)**
**Gender**	
Male	0
Female	40
Non- binary	2
Missing	4
**Age**	
18–24	1
25–29	7
30–34	10
35–39	3
40 −44	8
45–49	8
50–54	1
55–59	2
60–64	1
70–74	1
Missing	4
**Ethnicity**	
African Somali	4
Arab	1
Black/ Black British	1
Caribbean/Caribbean British	2
Pakistani/Pakistani British	8
White English, Welsh, Scottish, Northern Irish or British	19
Other White	3
Mixed or Multiple	3
Other	1
Missing	4
**Participants’ (home) local authority**	
Bury	11
Manchester	16
Oldham	1
Rochdale	2
Salford	4
Stockport	2
Tameside	2
Trafford	1
Wigan	1
Other	2
Missing	4
**Participants’ (home) deprivation score**^**bc**^	
IMD decile 1 & 2	18
IMD decile 3 & 4	6
IMD decile 6 & 9	5
Missing	9
**Religion**	
Muslim	12
Christian	13
No religion	14
Religion not stated	1
Prefer not to say	2
Missing	4
**Disability or pre-existing health condition (self-reported)**^d^	
Yes	15
No	28
Prefer not to say	1
Missing	4

^a^Demographic information was not received for 4 participants

^b^Based on available total 29 matched postcodes to IMD decile

^c^IMD decile based on total IMD score. Deciles ranked with lowest being the areas of highest deprivation

^d^Where participants self-reported as autistic, ADHD or other types of neurodivergent conditions, we have reported this using the term ‘neurodivergent’ to preserve anonymity

Participants from under screened groups were recruited to the study in two ways:

1. Via community groups and organisations

Contact details of relevant organisations were obtained from online searches and pre-existing professional networks. Organisations were then approached by the researcher via email explaining the purpose of the study and what collaboration involved. A request for help in recruiting individuals to the study was included in the email specifying that this could be by displaying a poster about the research or promoting a focus group with individuals who engaged with the organisation. All recruitment material for the study included the contact details of the researcher and interested individuals were asked to contact the researcher directly. Potential participants who contacted the researcher were emailed a participant information sheet.

Two community groups, a charity who supports people experiencing homelessness and a charity that supports women’s health in local communities supported recruitment and facilitated setup of the focus groups. In each case, potential participants were approached by the research team or staff from the voluntary or community organisation and provided with information sheets on behalf of the research team. Interviews (*n* = 3) were also conducted by the researchers on a mobile street-health unit run by a local charity and a ‘drop-in’ interview session was held at their premises in central Manchester, where two interviews were held. Informed written consent to participate was obtained prior to data collection, by two members of the research team.

2. GP Practices in the Greater Manchester area

We identified the GP practices in the Greater Manchester area that had the lowest cervical screening uptake, on average, between the financial years of 2013/14 to 2021/22. We used publicly available Quality and Outcomes Framework (QOF) data from NHS England [49]. These data include practice level information on the proportion of women between 25 and 64 years old who have been screened in the past 6 months. Once low uptake practices were identified we emailed them a study information letter. In addition, we distributed an expression of interest email via the NIHR Clinical Research Network (CRN) to the same practices, publicising the study and requesting that interested practices contact the research team directly. Two GP practices aided the study. One practice contacted eligible patients via text message, briefly describing the study and asking interested individuals to contact the researcher directly. The other practice contacted eligible patients via telephone and obtained their consent for the researcher to contact them using a ‘consent to contact’ form. Individuals who contacted the researcher directly or consented to the researcher contacting them were sent a participant information sheet via email.

Participants were reimbursed for their time with a shopping voucher.

### Data collection

Data collection took place between March and August 2023. Topic guides were developed, informed by the literature and subsequently reviewed by the project’s Advisory Group. Individuals from under-screened groups were asked open-ended questions about their experiences of cervical screening including interactions with HCPs. Topic guides are provided as supplementary material (see file 1). The researchers conducting the interviews provided participants with a basic verbal description of the two self-sampling methods (self-swabbing and urine testing) and asked participants’ for their initial impressions. They were then shown the self-sampling devices (vaginal self-swabbing and urine collection device) and asked again for their impressions. Participants were prompted to discuss their willingness to try either of the self-sampling methods, their preferred location (at home or at a GP surgery) for using the self-sampling methods, and their opinions on how the self-sampling methods might impact their experience of screening, which often included likelihood of attendance.

Participants were invited to participate in either a semi-structured interview or a focus group and were given the option of a face-to-face, online interview (via Zoom) or telephone call. Of the participants from under screened groups who took part, 16 participated via one of two in-person focus groups and 30 took part in individual interviews. Seven interviews took place face-to-face and the remainder took place online. Two focus groups were held in community spaces, and five interviews took place in a specialist community organisation who support women who undertake street sex work, including a mobile health unit. Interviews and focus groups were conducted by SG and HG[. The audio-recorded interviews were anonymised and transcribed verbatim.

### Data analysis

The qualitative data was analysed using an adapted framework method, where thematic analysis was used to inductively generate a set of themes from the data [50], [51]. This method produces a matrix of summarised data and provides a structure to analyse and summarise data within key themes [52]. The approach allows for analysis according to predefined themes and themes that emerge more inductively from the data [52, 53], drawing on feminist framings and discourses surrounding choice, autonomy, etc. Primary coding of the transcripts was conducted by SG, HG & KH, where the analytical framework was used as a starting point for coding initial transcripts structured by insights from the findings from collaborating team members, as well as key themes surrounding barriers to screening identified in the existing literature. The researchers and wider team and project Advisory Group worked closely together during all stages of analysis to discuss initial coding and to develop and refine the final themes. After analysis of all the transcripts and the final set of themes were identified, the access as human fit framework was retrospectively applied to help describe the data in the context of access, as it was apparent there was a wider and relevant context surrounding organisational, institutional and logistical factors that played a part in the barriers to screening, especially in regards to the potential implementation of self-sampling. An intersectional lens was also applied to the analysis: participants’ characteristics from the completed demographic sheets were analysed in tandem with the coding of the themes from the interviews/focus groups, alongside relevant contextual information in the transcripts to explore intersectional identities and personal experiences. Table 2 below outlines the key principles of the access of human fit, alongside the themes and sub-themes.

**Table 2:** Qualitative data themes & access as human fit

**Qualitative data: themes**	**Access as human fit**^a^
Interpersonal barriers with HCPs Communication with HCPs	Human factors (human interaction) Attitudes; Beliefs; Empowerment; Expectations; Experiences; Knowledge, Resources; Roles
Previous negative experiences	
Lack of information	Seeking health care; perception of needs and desire for care
Organisational level / practical barriers Issues booking Interactions with healthcare professionals	Workforce abilities < – system factors Reaching health care
Positive screening experiences Being made to feel comfortable Clear information	Human factors (human interaction) Attitudes; Beliefs; Empowerment; Expectations; Experiences; Knowledge Resources; Roles
Self-sampling methods	
Accessibility	Healthcare utilisation (workforce/system ability to be appropriate)
Inaccessibility	Healthcare utilisation (workforce/system ability to be appropriate)
Enhanced choice	Empowerment
Concerns over efficacy	Ability to be appropriate (Technical and Interpersonal quality; Adequacy)

^a^Adapted from Vourhees et al 2022

### Patient and public involvement & engagement

Patient and Public Involvement and Engagement (PPIE) was integrated in the project from the inception and design phase to ensure the project sufficiently reflected key contextual factors surrounding the design and data collection of the project. PPIE was undertaken during the development of the funding application stage via consultations over Zoom with Voluntary, Community and Social Enterprise (VCSE) partners, and throughout the design and implementation of the project via the inclusion of VCSE partners on the funding application, and the development of a PPIE Advisory group early in the project lifecycle. The Advisory Group was made up of diverse members of local communities in Greater Manchester, who advised on study design and setup including the research questions, language and topic guides . The Advisory Group also provided feedback on the key themes arising from analysis and will continue to be involved in disseminating the findings of the study.

### Ethical approval

The study was approved by NHS/HRA Research Ethics Committee on 1st February 2023 reference number 22/WM/0269.

### Findings

The findings below are outlined within three main themes: Barriers to screening: previous negative experiences and interactions with healthcare professionals; Positive experiences: appropriate communication, adequate information, individualised care; Views and responses to new self-sampling methods: empowerment, choice, autonomy and control.

### Barriers to screening: previous negative experiences and interactions with healthcare professionals

Patient experience, here in the context of previous experiences in healthcare settings and with screening particularly, is central to understanding access to cervical screening for under-screened groups. Many participants described previous negative experiences in the screening process as barriers to attending screening. These experiences included the uncomfortable nature of the examination and pain experienced, where attending screening was often discussed in this context. These points were raised universally across participant groups: from a range of minoritised ethnic and religious backgrounds, across different age cohorts, autistic participants and participants with mental health conditions, as well as those without health conditions. These points were also raised in the context of a personal and intimate examination, which consistent with feminist framings around bodily autonomy were perceived to be more sensitive because of this nature.

*….You’re touching my body, to me that’s very personal. It’s not just sort of, oh, whip your clothes off [name redacted], get on there, and out the room you go... I just think if I’ve had a bad experience, I’m sort of more likely to think, well, the last one I had, I left it another six months, I was umming and ah-ing whether or not to go.* (participant 25, White British, neurodivergent)

*I’m normally quite good at like gritting my teeth through something, but I was just fully in tears, it was so painful.* (participant 42, 25–29, White British)

Interpersonal interactions with healthcare professionals were raised by participants as linked to previous negative experiences, and this in turn linked to a reluctance to attend screening. Participants described being made to feel uncomfortable by un-empathetic HCPs, feeling rushed or having the examination rushed. Some participants were not comfortable with HCPs during the process because of a lack reassurance, not being put at ease and little or no communication about the process and what to expect. This was identified by mostly younger participants, participants from Arab, Black, White British, Pakistani, or multiple ethnic backgrounds, various religious background, and amongst participants with mental health conditions.

*….the second time, it was very much like in, out, goodbye. Oh, you’ve also got a cyst, but don’t worry about it. See you later.* (participant 38, female, 25–29, anxiety/depression)

*The woman who did it was, I felt, like, kind of, heavy handed and, like, quite insensitive, basically, not very personal and just, basically, she just, sort of, like, shoved the plastic thing in and did what she needed to do. And then, with barely any conversation or consideration for what was going on. Yeah, so I found that quite difficult*. (participant 19, 30–34, White British)

These experiences led to feelings of dehumanisation amongst some participants, reflecting feminist framings around disempowerment, and insinuating a lack of control over their bodies and of the choice to have screening done. This was driven by the interaction with HCPs who participants perceived lacked compassion towards patients’ fear and anxiety, especially towards the speculum. One participant describing having a panic attack during the examination.

*She had to do it quite a few times as well because of my…apparently my body was shifting around a bit…and because I knew that I just had to get it done, and so I didn’t feel comfortable being, like, actually stop, I don’t want to do this anymore…[ ] It just…I just didn’t feel like I had…literally as I walked out, I was like, oh, I really didn’t want to have done that, and I just didn’t feel comfortable voicing that... I think for me it’s…it made me think about the things that we’re asked to do and why we’re asked to do them….. …Obviously, I know that I’m…I’m just a number in a system in terms of, okay, let’s get this done…. I think for me, I don’t know if it’s something that I’ll get done…I’ll do again. Just because I don’t think an actual person is considered in the process, it’s just you just need to get this done, so we need to get this done.* (participant 39, 25–29, Black/Black British)

A significant element of discomfort by participants was related specifically to the speculum itself, and this was often discussed in the context of a reluctance to attend screening. Fear of the speculum was identified as a barrier to attending screening by participants who experience homelessness, participants with mental health conditions, autistic participants, participants from Pakistani, White British, African Somali backgrounds, Christian and Muslim faiths and across mixed age-groups.

*….the picture in your head that you’re going to have that speculum inside you. [ ] before they even put a speculum anywhere near you, you’re tensing your body up. And she’s like, what you doing that for, I’ve not even touched you yet. Because there’s a thought of it.* (participant 37, female, White British)

Despite the articulated barriers, participants were normalised to undergoing cervical screening, and many participants identified that despite barriers around pain, discomfort and negative interactions with HCPs, they still attended screening. For many, it was seen as compulsory, simply doing it because of a perceived necessity to their health. This indicated a lack of choice and it was raised that this obligation was aided by persistent communication and messaging in the forms of text or call reminders.

*“it’s just been sort of ingrained into me, it’s something you have to do… I feel it’s been something that I…I should have to go along just for the sake, because everybody else tells me I have to.”* (participant 25, White British, neurodivergent)

*I think I've never thought about it. Now that you’ve mentioned, I think I have never thought about it and I think for me it's something that I just have to do it, so even with the pain that I feel when the smear test is being done, I just have in the back of my mind, this is something I need to go through to have myself checked. So as quickly as it can be done, let's do it, and then we get over to the other side*. (participant 48)

### Positive experiences: appropriate communication, adequate information, individualised care

Conversely, nearly as many participants who described negative experiences (*n* = 19) described positive screening experiences (*n* = 12), with some describing both (*n* = 3). In these scenarios, HCPs communicated appropriately and provided adequate information, which was seen to be accommodated via an individualised approach. In these instances, participants described that they had been made to feel comfortable by HCPs, experienced good communication about the procedure, offered an explanation of what to expect during the examination, and were put at ease, resulting in a greater sense of control over the process. Participants (*n* = 12) who raised these points were neurodivergent, those from White British, Romanian, Pakistani, Caribbean backgrounds, women who undertook sex-work, and from Christian and Muslim faiths, across age groups. One woman described:

*…I have sensory issues, I’m not exactly sure how it’s going to go, or what you’re going to do. And she took out all the instruments, and the speculum and everything. And she talked me through it before she did it, and said, this is exactly what I’m going to do, I need you to sit like this. And she was really good at explaining everything, which really helped, yes, made me a lot less anxious. [ ] I said, well, I find these kinds of things really painful. So, she said, well, we’ll use a smaller one, we’ll try this, we’ll try that. [ ] And yes, she went through it step by step, before she did it. And then, when she was actually doing the smear test, [ ] she walked me through it, bit by bit, which made me feel so much more relaxed, because I knew exactly what was happening as it was happening…*(participant 23, White British, neurodivergent)

Participants highlighted the importance of communication prior to, during and after the appointment, where this communicates key information and heightens knowledge about cervical screening. For example, receiving appropriate information and explanations about the examination, and having results communicated. Practical issues such as ease of booking and the option of accessible appointments were noted.

*I do think it’s really good, how my practice [ ] send[s] out that information letter, of being able to bring someone and what to expect and stuff like that, outside of what the NHS has done, the GP sends that as well.* (participant 45, non-binary, 25–29, Black/Black British)

*Everything has been well explained and I’ve been asked if I need or want, you know, a chaperone or anything like that.* (participant 41, 25–29, neurodivergent)

### Views and responses to new self-sampling methods: empowerment, choice, autonomy and control

Overall, new self-sampling approaches to cervical screening were positively received across all participant groups. Self-sampling methods were seen to be more accessible than the traditional speculum method, because they were deemed to be less invasive, and less stressful to undergo, in part because they were self-administered and removed the necessary interaction with the HCP. Consistent with feminist framings around bodily control and autonomy, the self-sampling methods were seen to actualise principles of control and autonomy.

*It’s easier because you can do it all yourself, you don’t have to get someone else to do it and you’re more comfortable…*

*for me, a lot of the anxiety around the screening [ ]…is having somebody else do an invasive examination, [ ]…so, yeah, it would eliminate that, or it would just be me doing it, so it would be much better, yeah.* (participant 43)

*I'm doing it in my own privacy, so I would do it at home and then take the sample to the GP so it's not as intrusive as the method that I think is used right now is. So I feel it's more respectful as well.* (participant 48)

*[ ] I think it’s way more accessible, like, it means you can just do it in your own time at your own pace when you’re ready.* (participant 19, female, 30–34, White British)

A large part of positive views towards the self-sampling methods was due to the removal of the need for the speculum.

*…the speculum is literally horrible, like, that is horrible, it’s such a horrible, archaic looking device. [ ] So the lack of that sounds great,* (participant 38, female, 25–29, White British)

*they’re [the speculum] scary. They’re just…even just looking at them, they're just horrible, scary contraptions. …[the self-swab]…it's really simple, and looking at it, it doesn't scare me. Whereas, when they do it the traditional way, I know they use the speculum, but they use something similar. So, if you remove the speculum element of it, I think I'd be way more comfortable, yes.* (participant 23, 30–34, neurodivergent)

Participants who had or currently experience homelessness, women who undertake sex work, participants with mental health conditions, participants from White British, Pakistani/Pakistani British backgrounds, Christian and Muslim faiths, and across all ages of participants, especially highlighted the benefit of being able to do screening in the comfort of their own homes (in a safe and familiar environment). This environment was one which was seen to be more practical and convenient as it removed the need to attend a GP practice and interact with HCPs. It was highlighted that this ease of access may reduce associated barriers around anxiety etc. Conversely, three participants, those that experience or have experienced homelessness, White British, younger, would prefer to do the sampling at the GP practice. Four participants, from Black, White British, backgrounds between 40–49, would be happy to do the self-sampling either at home or at the GP practice.

*I think it’s a good idea, you’re doing it at home. Because, I suppose, people, their life is hectic enough and they don’t get to appointments and that would be ideal, wouldn’t it? I think it’s great because I’m on sedatives and I find it very hard to get to appointments and sometimes I miss them. Whereas if that’s sent to you, you can just do it in your own time and send it back. I think that’s a really good idea.* (participant 27, female, White British)

*You know, if you do in that home, you probably more likely to just quickly get that over and done with than all of the sort of nervousness you might have about going to an appointment somewhere…. I think it just makes it less of a big deal. It's not taking a huge chunk out of your day and you're not ringing up the doctors to make the appointment, and it removes a lot of steps of the process.* (participant 31, female, 18–24, generalised anxiety disorder & depression)

*the barrier isn’t the test, the barrier’s the way it’s done...It’s, kind of, I don’t think, well I know that, like, urine testing doesn’t bring up the same stuff that intimate examinations do.* (participant 32, female, 25–29, Caribbean)

In line with feminist perspectives around healthcare, ultimately what was articulated as the most significant factor was that self-sampling methods gave patients a choice of which method was most suitable for them; where to do it; and whether to do it themselves or have a HCP take the sample. Consequently, there was a sense that self-sampling methods would increase the propensity to attend screening amongst the groups sampled, consistent with feminist framings that champion inclusive approaches which reduce the homogenisation of people in their reproductive healthcare.

*I think giving everyone the option is important because everyone is different.* (participant 12, female 40–44, White British)

*It just feels like there should be more options for people, I’m sure lots of people will still do it the traditional way, but if they know that there is an alternative, I feel like you would get more of the fringe people to do it [ ]… the people at the edges, who are kind of like, pushed out because the traditional method is so difficult for them, I feel like they would be the ones who would probably use these alternative methods a lot more.* (participant 45 non-binary, Black/Black British)

Choice of methods was linked closely to control and autonomy. A lack of autonomy and control intertwined with a lack of choice, where the self-sampling methods were perceived to subvert the lack of choice and autonomy associated with the traditional screening method. This was highlighted by younger participants (34 and under), White British, Pakistani, Black participants, women who experience homelessness and participants with mental health and neurodiverse participants including autistic participants.

*I think being given the choice; you can make it yourself. Because now, it’s just like, you have to go to your GP, your GP has to do it for you, and you don’t really get a choice in the matter, someone’s going to touch you and someone’s just going to do it. Whereas, if you have the choice, then at least you can make the decision for yourself, how you want to do it, who’s going to do it, where you’re going to do it. I think that’s a big thing, yes.* (participant 23, female, 30–34, neurodivergent)

*I guess being able to do the test yourself, it’s like for something like this. It’s nice to have a bit of ownership over your health and the experience.* (participant 31, 18–24, White British)

*It gives you a bit more control over it yourself.* (participant 36, female, White British, mental illness)

*Just being in control of it, myself. What I don’t like is someone else having to do it and the, what did you call it, the speculum?* (participant 19, female, 30–34, White British)

Participants felt that choice of sampling method and location empowered them in the cervical screening process, and allowed them to gain a sense of control and ownership over taking part.

Most participants said they would feel confident using the self-sampling methods, emphasising familiarity with self-swabbing and urine sampling (for example, sexual health screening, UTI testing, for pregnancy etc.). However, some participants (from Pakistani , White British backgrounds, Muslim faiths and those with neuro-developmental conditions), were concerned about the usability of both self-sampling methods, citing apprehensiveness about dexterity.

*….for me, it’s [the urine sampling] too fiddly, too fiddly.* (participant 25, White British, neurodivergent)

*Now, that’s [the urine sampling] plastic. Yeah, that’s right. I mean, the whole sensation and the look of it is a little bit like, ooh daunting. So the swab is much more friendly, you know, it’s just like, oh yeah, I know how to use it, it’s alright.* (participant 30, female, 30–34, neurodivergent)

*[the urine sampling] looks a bit difficult to use, it’s not looking very simple.* (participant 44, female, 35–39, Pakistani/Pakistani British)

*It’s important to recognise that a lot of us have got fine motor and gross motor differences. And that first one [the self swab], I like the idea of doing a wee better, I would prefer to do the wee, but for me, that’s too many bits, that can go wrong, and I’d probably drop it in the toilet……with my fine motor differences.”* (participant 22, non-binary, 40–44, neuro-divergent)

*I think the [self-swab], I probably would find that uncomfortable because I don’t even know I would do it right. Even though it’s probably just taking the swab and doing it.* (participant 44, female, 35–39, Pakistani/Pakistani British)

*The swabbing one, I think I’d be a bit wary of that in case I do it wrong.* (participant 04, female, 55–59, Pakistani/Pakistani British)

For some, views towards the self-sampling methods were discussed within the context of the normalisation of the unpleasant smear test, where there was confusion and doubt around why self-sampling was not already offered.

*…I guess I’m surprised that you’re even [ ] saying that these are potentially possible because it’s always felt like the smear test has been, it’s got this really brutal procedure, with, like, big instruments and if you’re saying you can do it through urine and like a little tiny swab…* (participant 33, female, 35–39, White British)

*I’m a bit confused about why that’s not always been an option, if that’s possible?....I think, it’s just made me…if…if, I’m just a bit, still a bit confused about why, having…yeah, having had a really painful experience, about why that has ever been necessary, if it’s possible to just have a wee. Yeah, and like, I think, like I said before, just like reiterate, if that had been the option at the time, or if that was the option now, I would have done the test ages ago.* (participant 42, female, 25–29, White British*)*

Some participants asked questions about the accuracy of self-sampling in comparison to the traditional method, and raised concerns around their confidence in taking the self-sample.

*What if you don't get it deep enough inside you. Because to be able to detect the big C word, which is what smear tests are developed for, they actually know where to go, how far to go inside you to do the scraping…. [ ] am I touching the right place to be able to detect something? So, I'm going to say no to that one.* (participant 37, female, 45–49, White British)

*Tell me exactly how it works, like, there are cells in the urine that you check that are similar, like, how exactly? ….I mean, again, I’m not a scientist, I’m not a medical professional, but it’s just…I don’t know. It’s the curiosity of, like, how exactly it works at that microscopic level, how do you work it out? I don’t feel it’s kind of like too much, because usually with those leaflets, they try to tell you sometimes, like, what this is about and how…* (participant 30, female, 30–34, neurodivergent)

Here, scepticism surrounded the normalisation of traditional cervical screening tests:

“*I don’t know, it's just not sitting me with. Maybe because I’m too used to having the normal smear test at the doctors, I don’t know.” (*participant 37, female, 45–49, White British)

*The method is extremely different to what I experienced, so it would make me question why this method is completely different to what I experienced?* (participant 32, female, 25–29, Caribbean/Caribbean British)

In order for self-sampling to be perceived as a reliable alternative to traditional cervical screening, participants identified the need for accessible and appropriate information on the self-sampling methods. Participants suggested that this should include diagrams and video explainers of how to use the self-sampling methods, highlighting that written information alone would not suffice. Alongside this information, participants noted that the rationale for introducing self-sampling should be clearly communicated to patients. For instance, the accuracy of self-sampling methods and how they work should be clearly explained to inform patients about why these were being offered as an alternative to the healthcare practitioner-taken cervical sample.

*I think, for me personally, I find reading really difficult, so maybe an instructional video. Because I know you can get models, I don’t know what you call them, anatomical models, so maybe using one of those to show you exactly how to do it, and then, you can visually see, okay, this is what I need to do. Because reading all that information, it's not easy read, and a lot of the words they use are confusing. So, something like that would be really useful.* (participant 23, female, 30–34, White British, neurodivergent)

*I don’t see exactly how it works [ ] I’ll need more explanation about, like, how it works.* (participant 30, female, 30–34, neurodivergent)

*I mean, it will be helpful to see, you know, if I get a leaflet with the information or given the options beforehand. Like, I will expect kind of like a letter, invitation letter, saying, your screening is coming soon, and then probably a leaf…sorry, a leaflet inside. [ ], you have these options, this works at that amount, like, they work similarly, they have the same effectiveness or closer. [ ] like a chart, showing you the effectiveness. Probably also versus the regular cervical screening that I had before.* (participant 30, female, 30–34, neurodivergent)

## Discussion

Our findings demonstrate that known barriers to cervical screening persist for under-screened participant groups. These include barriers stemming from previous negative experiences of cervical screening, including discomfort and/or pain during the examination and fear of the speculum. Fear of the speculum examination was evident amongst all participant groups. Consistent with the principles of ‘access as human fit’ and situated in feminist perspectives, barriers pertaining to previous negative experiences centred around interactions with HCPs, including not being made to feel comfortable and a lack of empathy from HCPs, culminating in some cases where participants felt dehumanised and their needs unmet. These barriers were identified across participant groups, where such experiences were universally felt across different groups. These barriers are consistent with feminist readings of experiences of sexual and reproductive healthcare, where experiences associated with screening may be viewed as deprivileging women’s autonomy, homogenising and medicalising women’s bodies, dehumanising women in the process [41, 42]. These barriers also echo many of the well-known barriers identified in existing literature on the barriers to screening, demonstrating the enduring nature of such barriers amongst our participants.

Even though previously known barriers to screening persist amongst our sample of participants, numerous examples of good practice are evident. Many participants (including autistic and neurodiverse people, those from White British, Other White, Pakistani, Caribbean and Black backgrounds, women who undertake sex work, and from Muslim and Christian faiths, non-binary and younger groups) did identify instances where they had had positive experiences during the screening process. Here, they had received tailored, personalised care, and were met with appropriate communication, enabling their needs to be met during the process.

Whilst barriers to screening were described by nearly all participants across all participant groups, positive experiences were less commonly described (although nearly as many participants organically raised positive experiences as negative ones). Nevertheless, the variation of experiences within and between participant groups suggests that good practice is not currently implemented consistently for all under-served groups in our sample, with implications for intersecting experiences of marginalisation along specific axes of inequalities. For instance, there is a notable distinction between the prevalence of negative experiences versus positive experience along the lines of ethnicity, with disproportionate numbers of participants from a minoritised ethnic background reporting negative experiences. The fact that many participants in our sample attend screening despite the variation in their experiences, with notable prevalence of existing barriers (as opposed to new barriers not already identified in the literature) indicates inconsistencies in the implementation of and access to screening services in relation to human fit.

Interactions withHCPs can be understood as the central feature across both negative and positive experiences described by participants, indicating the importance of human interactions in the screening process as identified within 'access as human fit'. Interactional barriers are also identified in the wider literature on barriers to screening for participants amongst the general eligible population [24, 54]. Interactional barriers are also apparent within access to primary care and other healthcare services more generally [55, 56], reflecting the views of our sample who often referred to feeling rushed or under time pressure in screening appointments, and this is recognised in the wider literature around barriers to screening within the general population [24, 57]. However, these barriers are brought into sharper focus for under-screened participants, whereby intersecting identities and experiences of marginalisation may further impact the spectrum of fit of screening services vis à vis unmet need. This may be especially true for neurodiverse participants with sensory issues, and participants with mental health conditions, who may experience increased anxiety about the process and therefore require additional time or considered communication from HCPs. As such, it follows that these barriers may be more pronounced for under-screened groups, and may explain why these well-known barriers to screening remain amongst our sample (although it appears that this may not pre-determine screening attendance a priori for all groups in the study).

Nevertheless, our sample indicates a reasonably high level of screening coverage; 46% of the sample had had at least one screening test and could be considered ‘regular attenders’. A substantial proportion, just under a third, of the sample had never attended, had missed at least one, or were not planning to attend again. This may be explained through the ‘normalisation’ of screening, where despite significant and widespread negative experiences, attending screening remains normalised for many women, and may therefore explain the high level of attendance rates amongst our participants. This upholds feminist perspectives that describe that normalisation reflects a level of social conditioning, in which notions around the ‘duty’ to attend screening coincides with a moral obligation of preventative healthcare (whereby compliance is the ultimate goal [39]). This may, therefore, describe why women participate despite known barriers, and explains the normalisation of screening framed within discussions of both positive and negative experiences.

The variation in the data described above suggests there may be varying relational, inter-personal, and organisational capabilities and capacities in the precursory conditions which deem screening services accessible or inaccessible. On the supply side, the negotiation of interactions between patient and provider further illustrates the importance of the access paradox of unmet need (from patients) and those needs being met by the capabilities and capacities of the workforce [58]. Taken together with the variation in screening history amongst our participants indicates that access to screening may be seen as fluid and dynamic; situated on a spectrum of access between met and unmet needs of both the capabilities and capacities of the population to attend screening, and the workforce to deliver appropriate services that meet these needs, as described in the human fit model [44]. Indeed, such examples of differential patient experience in cervical screening suggests important constraining factors of the abilities of the healthcare workforce. The workforce may be institutionally and organisationally constrained to appropriately and adequately accommodate patients “within the context of available resources, system factors, and workload pressures” ( [44]p346). Such elements influence the ability to accept patients or be acceptable to patients, the ability to be available and to accommodate patients and to appropriately address the needs of patients [44]. These factors, in turn, impact patients’ ability to engage with and actively seek services, and may therefore explain the variation in experiences described here, supporting findings from a recent study that identifies the importance of key organisational factors (workforce levels and patient list size) in primary care as associated to screening coverage in English primary care [3].

Participants welcomed the introduction of self-sampling methods, with the majority of participants (across all participant groups) describing the benefits of self-sampling methods in comparison to traditional methods, including offering the choice of sampling method and location (i.e. at home or at the GP practice). This, along with the opportunity to self-sample was seen to make cervical screening less invasive, in particular, as these sampling methods removed the need for the speculum and examination from healthcare professionals, identified by younger participants (aged 25 - 34), participants from White British, Caribbean, Black British backgrounds, Christian faiths, and participants with neurodevelopmental disorders and mental health conditions. This supports existing evidence that suggests that under-screened groups may be more amenable to self-sampling methods if they have had negative previous screening experiences [57].

Ultimately, self-sampling was seen to provide greater empowerment and autonomy, enhancing control over the process through increased choice: of setting and method. The desire for and need to maintain control is known to impact women’s decisions and attitudes towards cervical screening, with the exertion of control articulated in both attending and abstaining from screening, where screening is perceived as a threat to an individual’s autonomous control [59]. As such, self-sampling methods may address some of the contextual and supply-side barriers which in turn impact on women’s choice to attend screening, as they are able to take greater ownership (underpinned by choice). Following Ells’ conceptualisation of informed choice [35, 60], accommodating a broader concept of autonomy may, in virtue of this, illicit greater opportunity for reimagined forms of power within the cervical screening programme itself.

Therefore, implementation of alternative screening methods may present the opportunity for a renewed sense of informed choice and empowerment, echoed amongst our participants that these self-sampling methods would, for some, increase their likelihood of being screened. The concept and opportunity of choice for patients accessing cervical screening services aligns closely with person-centred and individualised care, articulated through empowerment as key underpinning pillars of access as human fit model [44]p 346). The findings here and elsewhere in respect to screening [24] demonstrate the importance of personalised care which accommodates for individual needs, which is consistent with Voorhees and colleagues’ human fit model and supports feminist framings of issues relating to reproductive healthcare.

Our study did not reveal significant differences in preferences for self-sampling methods; however, this was not the primary focus of our research. Notably, all participants perceived self-sampling as a means of enhancing empowerment and autonomy, with both innovative methods addressing certain contextual and supply-side barriers that influence women's decisions to participate in screening. Nevertheless, some participants identified risks that may undermine the new self-sampling methods’ fit. Some participants (from Pakistani, White British backgrounds, Muslim faiths and neurodiverse participants) raised concerns about the usability of the self-sampling methods, pertaining to dexterity, ease of use and accuracy. While our findings suggest that both methods should be made available, we acknowledge that further investigation in this area could enhance the successful implementation of these new interventions. As such, self-sampling must be implemented alongside effective and accessible information and communication, including detailed and accessible information on how to use self-sampling, the accuracy of self-sampling and so on. Again, this emphasises the importance of the ‘fit’ of screening services, whereby the implementation of self-sampling as part of screening services are as important as the method itself, and is a central part of the choice provided to women.

To fully realise the identified benefits around choice, and empowerment, appropriate information must be addressed in their implementation, as knowledge may inform women’s participation in screening, potentially subverting power structures in cervical screening process [35, 43]. In addition, within this context of greater choice, there was a scepticism towards self-sampling methods centred around the normalisation of the traditional smear tests, where some participants were confused about the self-sampling methods and how different they were to traditional methods, resulting in some scepticism towards self-sampling. To help counteract this sceptism, self-sampling could be framed to the public in terms of research breakthrough and progress, recognising the need for more choice over screening methods as being responsive to patients’ needs and existing barriers.

Overall, how self-sampling methods are implemented will be key to their success. Their implementation will need to address prominent concerns and questions around efficacy, and provide sufficient information about how they work, to mitigate the effects of the normalised acceptance of traditional methods. One European study (across ten countries) highlights that information in cervical screening invitation letters are poor and biased, not enabling those invited to make an informed decision about attending screening, and another study in Estonia indicates that personalised communication is preferred and the translated material is especially important for minority groups [61, 62]. Thus, information on self-sampling methods should be targeted appropriately to different under-screened groups, with the most appropriate communication channels and mechanisms utilised (e.g. translated materials and dissemination via the voluntary sector to enhance reach). This otherwise risks the legitimacy of new self-sampling methods being accepted as a viable alternative to the traditional speculum method, meaning an enhanced choice for women may be not sufficiently realised.

### Strengths and limitations

Strengths of this study include the diverse sampling of participants achieved across a number of participant characteristics, including deprivation, ethnicity, religion, age etc. In addition, the study is timely due to the recent and ongoing focus of alternative screening methods including self-sampling, in which this study contributes to the evidence base for the implementation of self-sampling methods. Within the remit of this study (or similar qualitative study types), it is not possible to ascertain (with certainty) how intention may lead to behaviour change regarding propensity to use self-sampling. Nonetheless, we maintain that our findings reflect a meaningful representation of intent/behaviour resulting from the nature of the interview discussions where this was discussed in detail. Whilst our focus was on under-screened groups, the majority of the sample had previously attended cervical screening, resulting in an under-representation of participants who had never attended screening. Further research is needed to explore barriers amongst never-attenders, especially views towards self-sampling. Whilst this is a limitation in terms of an under-representation of perspectives of these groups reflected in the research findings, this gives our sample a unique perspective to compare the self-sampling methods to the traditional speculum, which, as the findings demonstrate, is a significant feature of self-sampling and the comparisons between traditional methods and self-sampling methods, fundamentally important.

## Conclusions

Barriers to cervical screening can be conceptualised within a framework of ‘access as human fit’ and feminist framings around control and body autonomy. In the context of access to screening for under-screened groups, interactional factors with healthcare professionals reconstitute current barriers to screening for under-screened groups, upholding and maintaining well-known barriers, but are also evidenced here to facilitate positive patient experience. Variation in patient experiences demonstrated amongst our participants highlights inconsistencies in how screening services are implemented and experienced for these groups, which may have implications for intersecting inequalities and further marginalise groups within the screening process. New self-sampling methods (vaginal swabs and urine sampling), may address some existing barriers through the proponents of enhanced choice leading to greater empowerment. Choice and empowerment is a central feature of self-sampling, and may better align to feminist understanding of healthcare services, where the introduction of new self-sampling methods may address some of the existing barriers identified here and elsewhere in the literature, around individualised approaches centring around choice, the speculum itself, the invasive nature of the examination, and practical barriers surrounding convenience and time restraints of attending GP practice appointments. An intersectional lens demonstrates the importance of understanding nuances in the perspectives of the study participants towards the barriers to screening and towards self-sampling methods, illustrating that a ‘one size fits all approach’ must be steered clear. Along this line, this is strongly caveated around the implementation of these new self-sampling methods, which will need to address key considerations around appropriate information and sufficient communication. Failure to implement self-sampling without adequate consideration and due reconciliation of these risks may threaten to undermine the identified and important benefits of self-sampling methods. The use of three theoretical frameworks which we have integrated into an overall lens for this analysis applies a new such perspective for examining the barriers to screening and in the context of new self-sampling methods, allowing for existing barriers to be examined and reflected on in a way not previously explored.

## Supplementary Information

Supplementary Material 1.

Supplementary Material 2.

## Data Availability

Data for this research data will not be made publicly available as individual privacy could be compromised. Please contact Stephanie Gillibrand (stephanie.gillibrand@manchester.ac.uk) for further information.

## Abbreviations

ADHD: Attention deficit hyperactivity disorder
HCPs: Healthcare professionals
CRN: NIHR Clinical Research Network
PPIE: Patient, Public, Involvement and Engagement
QOF: Quality Outcomes Framework
VCSE: Voluntary, Community and Social Enterprise

## Footnotes

1 Brand name is ‘Colipee’.

2 One participant had not yet been offered screening as they not eligible based on age.
