# Supplementary material for: Exploring the barriers to cervical screening and perspectives on new self-sampling methods amongst under-served groups
Source: BMC Health Serv Res. 2025 Jan 15;25:79. doi: 10.1186/s12913-024-12098-2 (PMC11734453; doi:10.1186/s12913-024-12098-2)
Supplement: Supplementary file 1 — Supplementary Material 1. [file 12913_2024_12098_MOESM1_ESM.docx]

**Semi-structured interview guide – FOR PATIENTS/PUBLIC**

*Introduction to participant*

*The following questions are guiding questions only. There is no expectation for you to discuss anything you do not wish to.*

*You may pause or stop the interview at any point with a justification.*

- Are you aware of smear tests? Have you been asked to get your smear test done?
- We would like to invite you to tell us about your experiences before, during and after the screening process? You are not expected to talk about anything you do not want to.

*Prompts*

*Emotive response I.e. fear; access barriers including lack of information on smear test,*  *accessibility, language of information; logistical barriers; trust; cultural barriers*

- Is the interaction with HCPs important during the screening process? Before, during and after the examination? *(here we mean healthcare professionals including administrative staff in the GP surgery, the GP and nurse, etc)*
  - *Can you tell us about your interactions with healthcare professionals before, during, and after the screening process?*
- Is the gender of HCPs a factor in the screenings process? Have you previously been given a choice ?

*Additional questions*

- Do the attitudes of healthcare professionals impact your decision to participate in screening?

**Alternative methods**

*Recently researchers have been working to develop two new alternative methods for smear tests.*

1. *Self-swabbing*
2. *Urine sampling*

*Prompts*

*Traditional ‘smear tests’ involve a speculum inserted into the vagina by a qualified nurse to take a sample which tests for high-risk human papillomavirus (HPV) that can cause cervical cell changes that may develop into cervical cancer. These new methods are ‘do-it-yourself' alternatives to that.^[[1]](#footnote-1)^*

*Self-swabbing – a ‘covid-test’ for ‘down there’; urine sampling ‘peeing in a cup’*

- What is your initial response/reaction to these new methods?
- Would you be open to trying these new methods? Either or both?
- How might these new methods change the screening process for you?
- Would you have a preference as to whether these new methods are done at home or during an appointment at the GP?

1. Factsheet that can be provided to participants if they require more detail post interview? <https://www.jostrust.org.uk/sites/default/files/downloads/cervical_screening_download_march_2020.pdf>

   coli-pee video <https://sites.manchester.ac.uk/aces/>

   <https://www.gov.uk/government/publications/cervical-screening-description-in-brief> - in different languages

   <https://www.jostrust.org.uk/information/hpv/how-do-people-get-hpv> about HPV [↑](#footnote-ref-1)
